# Computational model of blood flow in the aorto-coronary bypass graft

**DOI:** 10.1186/1475-925X-4-14

**Published:** 2005-03-04

**Authors:** Meena Sankaranarayanan, Leok Poh Chua, Dhanjoo N Ghista, Yong Seng Tan

**Affiliations:** 1 School of Mechanical and Production Engineering, Nanyang Technological University, 63 97 98, Singapore; 2 Bioengineering Division, Nanyang Technological University, 63 97 98, Singapore; 3 Department of Cardiothoracic Surgery, National Heart Centre, 16 87 52, Singapore

## Abstract

**Background:**

Coronary artery bypass grafting surgery is an effective treatment modality for patients with severe coronary artery disease. The conduits used during the surgery include both the arterial and venous conduits. Long- term graft patency rate for the internal mammary arterial graft is superior, but the same is not true for the saphenous vein grafts. At 10 years, more than 50% of the vein grafts would have occluded and many of them are diseased. Why do the saphenous vein grafts fail the test of time? Many causes have been proposed for saphenous graft failure. Some are non-modifiable and the rest are modifiable. Non-modifiable causes include different histological structure of the vein compared to artery, size disparity between coronary artery and saphenous vein. However, researches are more interested in the modifiable causes, such as graft flow dynamics and wall shear stress distribution at the anastomotic sites. Formation of intimal hyperplasia at the anastomotic junction has been implicated as the root cause of long- term graft failure.

Many researchers have analyzed the complex flow patterns in the distal sapheno-coronary anastomotic region, using various simulated model in an attempt to explain the site of preferential intimal hyperplasia based on the flow disturbances and differential wall stress distribution. In this paper, the geometrical bypass models (aorto-left coronary bypass graft model and aorto-right coronary bypass graft model) are based on real-life situations. In our models, the dimensions of the aorta, saphenous vein and the coronary artery simulate the actual dimensions at surgery. Both the proximal and distal anastomoses are considered at the same time, and we also take into the consideration the cross-sectional shape change of the venous conduit from circular to elliptical. Contrary to previous works, we have carried out computational fluid dynamics (CFD) study in the entire aorta-graft-perfused artery domain. The results reported here focus on (i) the complex flow patterns both at the proximal and distal anastomotic sites, and (ii) the wall shear stress distribution, which is an important factor that contributes to graft patency.

**Methods:**

The three-dimensional coronary bypass models of the aorto-right coronary bypass and the aorto-left coronary bypass systems are constructed using computational fluid-dynamics software (Fluent 6.0.1). To have a better understanding of the flow dynamics at specific time instants of the cardiac cycle, quasi-steady flow simulations are performed, using a finite-volume approach. The data input to the models are the physiological measurements of flow-rates at (i) the aortic entrance, (ii) the ascending aorta, (iii) the left coronary artery, and (iv) the right coronary artery.

**Results:**

The flow field and the wall shear stress are calculated throughout the cycle, but reported in this paper at two different instants of the cardiac cycle, one at the onset of ejection and the other during mid-diastole for both the right and left aorto-coronary bypass graft models. Plots of velocity-vector and the wall shear stress distributions are displayed in the aorto-graft-coronary arterial flow-field domain. We have shown (i) how the blocked coronary artery is being perfused in systole and diastole, (ii) the flow patterns at the two anastomotic junctions, proximal and distal anastomotic sites, and (iii) the shear stress distributions and their associations with arterial disease.

**Conclusion:**

The computed results have revealed that (i) maximum perfusion of the occluded artery occurs during mid-diastole, and (ii) the maximum wall shear-stress variation is observed around the distal anastomotic region. These results can enable the clinicians to have a better understanding of vein graft disease, and hopefully we can offer a solution to alleviate or delay the occurrence of vein graft disease.

## Background

**Figure 1:**
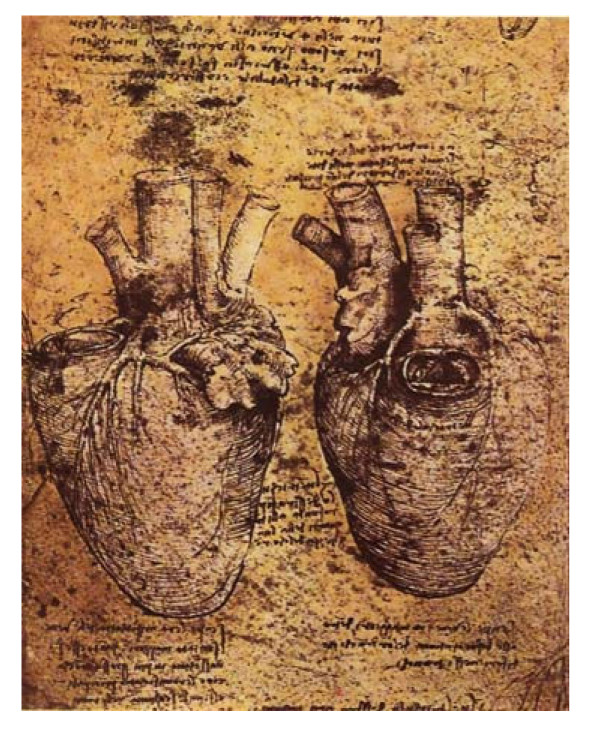
Leonardo da Vinci's sketches showing the distribution of the heart and blood vessels.

The complex anatomy of coronary vessels has made the investigation of coronary flow and hemodynamics one of the most difficult and challenging studies until today. Leonardo Da Vinci (generally called as "man ahead of time"), developed insightful sketches (some 550 years ago) of the anatomy of the heart and its blood vessels, as shown in Figure 1.

The major cause of death in both the developed and developing countries is cardiovascular disease. The study of coronary arterial circulation is important, because it is crucial in maintaining heart perfusion and function. The process of arteriosclerosis involves the formation of atherosclerotic plaques in the coronary tree. With time, this results in stenosis of the blood vessel and in turn decreases coronary flow. When it cannot match the oxygen demand of the myocardium, it results in ischemia and thereafter in an infarct. In order to overcome this problem of diminished perfusion of the affected myocardium, CABG is one of the treatment options that are being used to improve coronary perfusion. In this procedure, new routes around narrowed and blocked arteries are constructed with both arterial and venous conduits, allowing sufficient blood flow to be delivered to the ischemic heart muscles.

In spite of CABG being an effective surgical technique to revascularize the myocardium, 20–50% of bypass grafts fail due to the formation of intimal hyperplasia [1-3]. Previous studies have reported anastomotic intimal hyperplasia thickening to be associated with several factors, such as hemodynamic factors [4], compliance mismatch between graft material and host artery [5], biomaterials incompatibility [6]. For considering the influence of hemodynamic factors on graft patency, it is important to replicate the in-vivo geometry of the entire bypass conduit. In other words an arterial bypass flow model must be anatomically accurate, so as to capture the essential hemodynamics of the true geometry [7].

### Relation between wall shear stress and intimal hyperplasia

Flow regimes in end-to-side anastomosis, provide pertinent information concerning wall shear stress gradient which affects intimal hyperplasia thickening. The three- dimensional experimental studies of flow in an end-to-side anastomosis by Ojha et al [8] have revealed a relation between low and fluctuating wall shear stress and intimal hyperplasia. For the distal end-to-side anastomosis model, Hughes and How [9] have shown that intimal hyperplasia occurs in regions of flow separation at the toe and the heel, and that flow-stagnation is observed on the floor of the anastomosis. Additionally, animal model studies, conducted by Bassiouny et al [10], provide correlation of regions of low wall shear stress with areas of intimal hyperplasia.

### Using CFD for wall shear stress determination

Although wall shear stress distribution is a major factor in the onset of coronary diseases it cannot be measured directly and is hence calculated from velocity profiles. Even though these velocity profiles can be obtained in vivo using magnetic resonance (MR) or color-flow Doppler ultrasound (CDU), there are some limitations to measurements due to the small dimensions of the arteries. Thus the alternative to measure the wall shear stress is to use CFD to simulate flow in CABG geometry and thereafter compute the wall shear stress.

### Anastomosis geometry

Unfortunately, most of the modeling work in graft-flow is limited to only a part of the total bypass conduit geometry, namely the anastomosis site. As regards to the anastomosis geometry, Song MH et al [11] developed a Y-Figure anastomotic model for proximal arterial stenosis (at angles ranging from 10° to 30°), in order to analyse the three-dimensional simulation of coronary artery bypass grafting. In this work, in the end-to-side anastomosis model, all the vessels were adopted to be of the same diameter. The simulation results suggest that the more acute the angle of anastomosis, the smaller is the energy loss.

Even three-dimensional CFD simulations have been performed on end-to-side anstomosis of a stenosed coronary bypass, by Bertolotti and Deplano [12]. Herein, the anastomosis geometry was based on the assumption that the graft and the host vessel are of the same diameter, with the graft inclined at 45° to the host vessel. The inputs into the anastomotic domain were flow-rates from the graft and from the occluded proximal artery. The flow features were compared for different flow rates and distance of the anastomosis from the site of occlusion. It was concluded that the risk of intimal hyperplasia could be minimized if the anastomosis was sutured at a sufficient distance of length.

### Proximal artery flow conditions

An important factor is the effect of proximal artery flow condition on the hemodynamics at the distal end-to-side anastomosis. This effect has been analysed by Kute and Vorp [13], using CFD through the idealized geometry model consisting of equal diameter vessels with the graft inclined at 30°. The boundary conditions for the anastomotic model involved blunt velocity profiles into the graft and from the proximal artery, with a fixed pressure at the distal artery outlet. The velocity vectors for all the proximal arterial flow conditions exhibited skewing towards the floor of the artery, and their distribution varied with the flow conditions in the proximal artery.

### Graft flow determination

The combined use of imaging techniques and CFD may be necessary for meaningful clinical studies. With the availability of imaging techniques like coronary angiography, magnetic resonance angiogram (MRA), computed tomography (CT), several researchers have analysed the flow in realistic bypass graft models. There have also been some studies combining experimental measurements and CFD analyses [14-17].

Doppler analysis is generally preferred since it is safe, accurate and rapid when compared to angiography. Even though angiography certainly gives a definite answer about graft patency, there is more risk involved and it is time consuming and requires the assistance of a cardiologist. Doppler methods have been used by Bertolotti et al [18] and Lin et al [19] for graft flow analysis during minimally invasive CABG. The functioning of the graft has also been analysed through methods such as intraoperative angiography, probing of the anastomosis, electromagnetic flow meter, and the transit time flow measurement [20-22].

### Limitations of works carried out

It is clear from literature review that an important step towards realistic flow simulations concerns the generation of appropriate coronary vasculature geometry (involving the entire flow domain from the aorta to the perfused artery) with physiological boundary conditions. Unfortunately, a lot of work in these areas has been carried out on subsections of this bypass flow domain [8,9,11-13,15,16,18], and that too involving idealized geometry of the anastomosis section. Therefore in our work, we have addressed this drawback by studying the flow characteristics (by means of a three-dimensional CABG model) in the complete bypass domain under representative physiological conditions. We have thereby analysed realistic physiological simulations, and highlighted the resulting flow patterns and wall shear stress that are deemed to play a major role in intimal hyperplasia.

## Methods

### Geometrical Model

**Figure 2:**
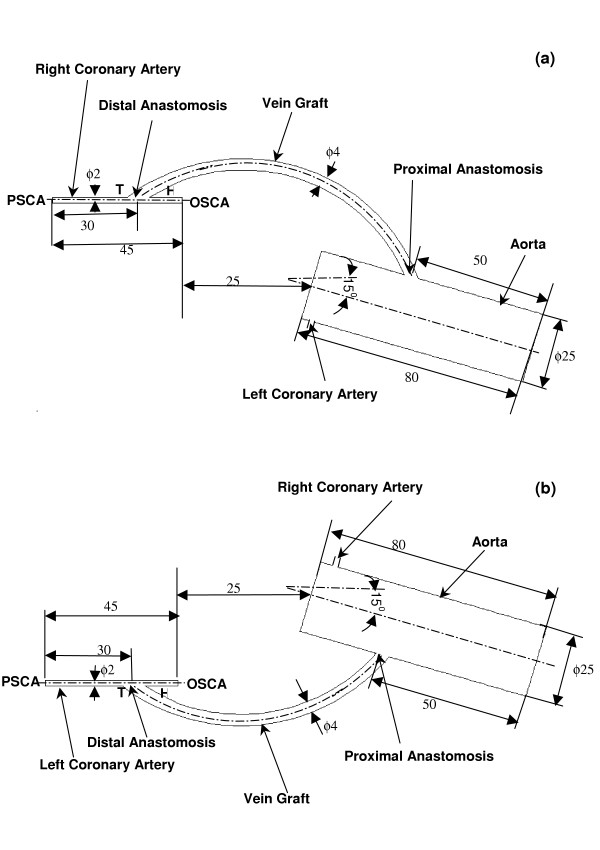
Geometry (plane view) and dimensions (in mm) of the bypass models of (a) The aorto-right coronary artery bypass model. (b) The aorto-left coronary artery bypass model. (PSCA-Perfused Segment of the Coronary Artery; OSCA-Occuluded Segment of the Coronary Artery; T-Toe; H-Heel)

The bypass models simulating the flow field of the anastomosis in the right and left aorto-saphenous bypass grafts are illustrated in Figures 2a and 2b respectively. The ascending aorta is modeled to be of length 80 mm with diameter 25 mm. The normal coronary artery is modeled to be of circular cross-section. The 100% occluded coronary artery is modeled as a straight tube, with dimensions of length 45 mm and diameter 2 mm.

In Figures 2a and 2b, the venous graft is shown to have a non-uniform circular cross-section, larger than that of the coronary artery. The intersection between the graft and the coronary vessel has an elliptical shape, which is due to the deformation of the larger diameter graft due to its suturing to the smaller diameter coronary vessel surface. The dimensions are provided by our cardiac surgeon joint-author (TYS) and the model has been constructed with this data.

### Model Assumptions, Data Input, and Boundary Conditions

**Figure 3:**
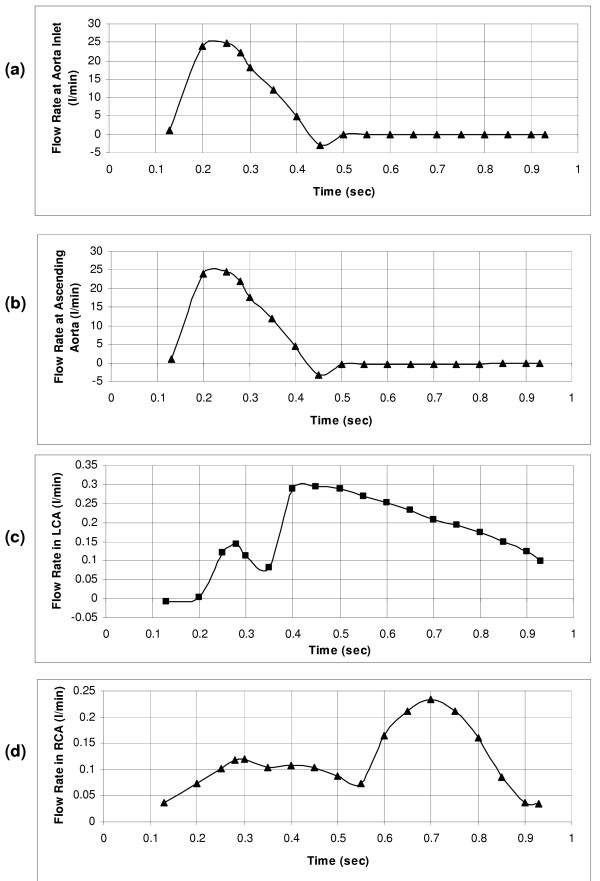
(a) Flow-rate waveform at the inlet of aorta. (b) Flow-rate waveform of the ascending aorta, reverse flow seen due to 4% of the stroke volume that goes into the ascending aorta. (c) Flow-rate waveform at the left coronary artery, derived from the flow velocity waveform obtained using intravascular Doppler ultrasonic flow technique. (d) Flow-rate waveform imposed at the right coronary artery, measured using a Doppler flow meter catheter.

The blood is assumed to be incompressible, with a Newtonian behaviour having dynamic viscosity (*μ*) of 0.00408 Pa and a density (*ρ*) of 1050 kg/m^3^. The blood vessel walls are assumed to be rigid and impermeable. For a quasi-steady, three-dimensional and laminar flow, the Navier-Stokes equations (for mass and momentum conservation) governing fluid motion is written as follows:





where  denotes the velocity vector in three dimensions. The distributions of velocity and wall shear stress are obtained by computationally solving the above equations subject to the boundary conditions given below. In accordance with equation (2), the flow field is automatically updated during each time-interval, by adopting the time-varying input data of the aorta (Figures 3a &3b), the left coronary artery (Figure 3c) and the right coronary artery (Figure 3d).

#### Systolic phase

This flow is obtained by prescribing a uniform velocity (across the cross-section) at the inlet to the aorta, from the left ventricle (LV). The velocity magnitude is computed from the physiologically representative stroke volume over the ejection period, based on the flow wave form [23] shown in Figure 3a. Through out this period, the left ventricle contracts and squeezing the coronaries, which are embedded in the cardiac muscle. This allows very little amount of blood to flow into the coronary arteries.

During systole, the inputs to the model consist of (i) monitored time-varying flow-rate waveform at the inlet of the aorta, (depicted in Figure 3a), adopted from Ganong [23], as well as (ii) monitored time-varying flow-rate waveform at the left coronary artery(depicted in Figure 3c) obtained from in-vivo intravascular Doppler ultrasonic technique [24], and (iii) monitored time-varying flow-rate waveform imposed at the exit of the right coronary artery (depicted in Figure 3d) obtained by means of a Doppler flow-meter catheter [25].

#### Diastolic phase

At the start of diastole, there exists a small amount of back-flow into the left ventricle through the aortic inlet (Figure 3a). For the rest of the diastolic phase, the aortic valve remains closed. In all these cases, blood flows back from the ascending aorta into the coronary arteries. Herein, we take into account that a major portion (second/third) of the backflow into the ascending aorta, (flow waveform displayed in Figure 3b) goes into the left coronary artery (Figure 3c), allowing the remainder to flow into the right coronary vessel (Figure 3d). This yields the blood volume flowing into the left-coronary vessel (during a cardiac cycle) to be 2.67% of the stroke volume, with the remaining 1.33% of the stroke volume going into the right coronary artery. From these specified flow-rates at the left coronary artery, and at the right coronary artery, the backflow from the ascending aorta into the aortic root domain is calculated.

Hence the data input to the model consists of (i) the calculated uniform velocity at the ascending aorta, and (ii) the flow conditions at the left and right coronaries obtained from the monitored time-varying input flow-rate waveforms (Figures 3c &3d).

### Fluid Dynamics Simulation Setup

The fluid dynamics simulations are performed by using a control-volume-based technique, implemented in the computational fluid dynamics (CFD) code Fluent [26]. The computation procedure of the commercial code consists of (i) construction of the geometry using a pre-processor, Gambit [27], (ii) meshing the computation domain, (iii) assigning boundary conditions in terms of velocities and flow-rate weightings, (iv) assigning fluid properties, and (v) the solution algorithm.

**The geometry of the aorto-coronary bypass graft models **is constructed in Gambit, using the dimensions provided by our cardiac-surgeon joint-author (TYS). The elements employed to mesh the computational domain consisted primarily of regular structured hexahedral elements as well as wedge elements wherever necessary. In order to carry out the mesh sensitivity analysis, numerical simulations were carried out by varying the number of mesh elements in the computational domain. Initially, the domain was discretized into 120974 hex/wedge elements. The accuracy of the simulation results was then improved by employing a finer mesh that contained 419765 elements. This number was further increased to 623138, but resulted in no significant improvement in the results. Thus to maintain a balance between the computational cost and the numerical accuracy, we concluded (based on the mesh independence test) that the appropriate number of elements for our study is 419765.

**In the solution algorithm used by Fluent**, the governing equations are solved sequentially. Because the governing equations are non-linear (and coupled), several iterations of the solution loop need to be performed before a converging solution is obtained. Using this approach, the resultant algebraic equations for the dependent variables in each control volume are solved sequentially by a point implicit (Gauss Seidel) linear equation solver, in conjunction with an algebraic multi-grid (AMG) method. The governing equations are solved iteratively until convergence of all flow variables is achieved. The solutions of all the flow variables are deemed to have converged once their residuals computed from two successive iterations are below the set desired convergence criteria of 10^-3^. The study was also carried out by setting the convergence criteria as 10^-4^; this did not alter the results obtained earlier.

## Results

For these numerical simulations we have incorporated the unsteady flow character by dividing the cardiac cycle into a number of small time interval, and analyzing for steady flow within these time intervals. To observe the velocity distribution features of the entire flow field, the computed velocity vectors are illustrated in the plane of symmetry at two different instants of the cardiac cycle.

### Simulated flow field for the aorto-right coronary bypass (Figures 4 and 5, for t = 0.13 sec and t = 0.7 sec respectively)

**Figure 4:**
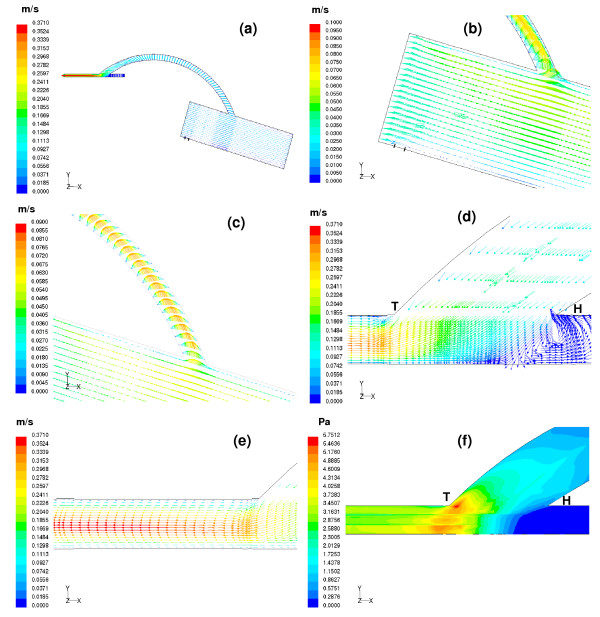
(a) Velocity vectors computed at t = 0.13 sec are depicted on the centre plane of the aorto-right coronary bypass model. (b) Enlarged view of the velocity vectors inside aorta. At the start of ejection, blood from the aortic inlet flows into the ascending aorta. The blood flow at a distance of 10 mm from the entrance of the aorta behaves almost like an inviscid flow. Very little amount of flow enters the coronaries; this may be due to the high pressure in the myocardium. (c) Parabolic profiles of the velocity vectors are observed inside the graft. Slight skewing of the flow profiles is seen due to the influence of the graft curvature. (d) A close view of the recirculation region in the artery-graft junction. Major portion of the flow exiting from the graft moves towards the distal portion of the right coronary vessel. (e) The flow pattern shows slight skewing towards the floor of the artery. With increasing distance skewing disappears thus shifting the maximum velocity magnitude 0.37 m/s to the centreline of the host artery. (f) A wide variation in the wall shear stress is observed at the distal anastomotic region. The weak recirculation zone at the proximal portion of the bypassed vessel results in negligible wall shear stress with the peak wall shear stress magnitude, 5.75 Pa appearing at the floor of the artery.

**Figure 5:**
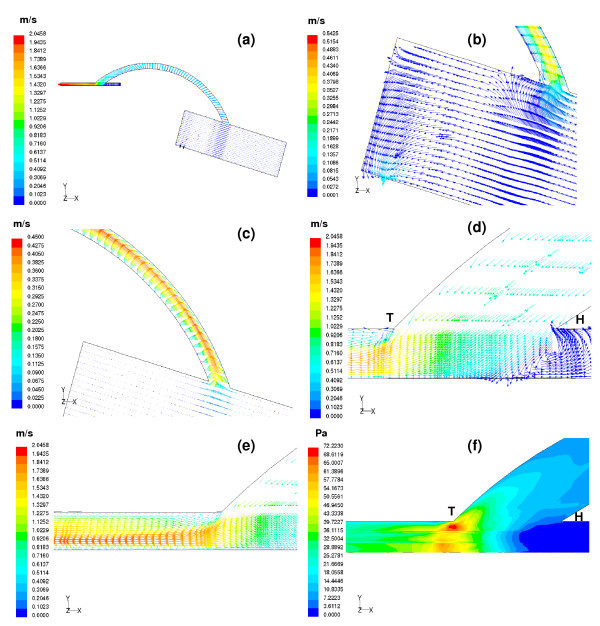
(a) Velocity vectors computed at t = 0.7 sec are depicted on the centre plane of the aorto-right coronary bypass model. (b) At the mid-diastolic instant, the aortic valve is fully closed and thus back flow from the ascending aorta enters both the coronary vessels, the left coronary vessel and the bypassed right coronary vessel. (c) Parabolic profiles of the velocity vectors are seen inside the graft. Maximum perfusion of the graft occurs at mid-diastole. (d) Maximum flow velocity approaching the graft exit is around 0.95 m/s. The flow exiting the graft with a higher velocity results in a stronger impingement on the floor of the artery. The strong recirculation region seen at the proximal portion of the distal anstomotic region forces the flow to move towards the right coronary artery exit. (e) The inner wall of the host artery exhibits significant skewing of the velocity profiles. The maximum flow velocity magnitude, 2.04 m/s, seen close to the floor of the artery is shifted to the centre line of the vessel with increasing axial distance. (f) Maximum perfusion occurs at the mid-diastolic instant, t = 0.7 sec. This gives rise to maximum wall shear stress with the peak magnitude being 72.22 Pa.

#### (a) Flow field in the aorto-right coronary bypass model at the onset of ejection, t = 0.13 sec

At the onset of ejection, at t = 0.13 sec, Figure 4a depicts the distribution of velocity-vectors in the flow field. The flow-velocity distributions in different sections of the flow field are depicted in Figures 4b–4e. The uniform velocity at the inlet to the aorta is 0.035 m/s. At a distance of 10 mm from the entrance, the computed flow field appears to be like inviscid flow. There is hardly any flow entering the graft at the start of systole as shown in Figure 4c. Figures (4d &4e) depict the flow-velocity distribution in the anastomosis domain. The flow pattern in the graft anastomed to the aorta at a distance of around 40 mm from the aorta entrance is parabolic, with very slight skewing. The forward flow coming from the graft into the host artery shows a small region of recirculation at the heel of the anatomosis domain (i.e. where the occluded (proximal) bypassed coronary artery connects to the anastomosis domain) as indicated in Figure 4d. The flow coming into the perfused right coronary vessel gets fully developed, with a peak velocity of 0.37 m/s, as depicted in Figure 4e.

The computed wall shear stress (product of velocity gradient at the wall and the viscosity of fluid) is depicted in Figure 4f as (i) a low wall shear-stress region in the heel section of the anastomosis where there is flow-stagnation along the floor of the artery and (ii) a high wall shear-stress at the toe of the anastomosis (associated with the disturbed flow patterns, shown in Figure 4d) which is prone to intimal hyperplasia.

#### (b) Flow field in the aorto-right coronary bypass model at the mid-diastolic instant, t = 0.7 sec

At the mid-diastolic instant (at t = 0.7 sec), when the aortic valve is closed, Figure 5a illustrates the velocity vector plots. Some amount of the backflow coming from the ascending aorta enters into the graft, and the rest goes into the left coronary vessel as shown in Figure 5b. Along the graft, the peak velocity is skewed towards the outer wall. Initially the flow is seen to follow the outer wall, and then this slowly turns to the centre as demonstrated in Figure 5c.

In the anastomosis domain, a strong region of recirculation is observed near the occluded end of the artery, which forces the flow to move into the perfused right coronary (distal) artery as indicated in Figure 5d. The flow in the perfused distal artery exhibits significant skewing of the velocity profiles towards the inner wall of the perfused (distal) artery, with a peak magnitude of 2.04 m/s as seen in Figure 5e.

The flow pattern variations give us insight into the wall-shear distribution. The high velocity gradients in the anastomosis give rise to large spatial variations in the resulting wall shear stress. The largest value of wall shear stress is seen in Figure 5f, near the toe of the graft-artery junction.

### Simulated flow field for the aorto-left coronary bypass (Figures 6 and 7, for t = 0.13 sec and t = 0.7 sec respectively)

**Figure 6:**
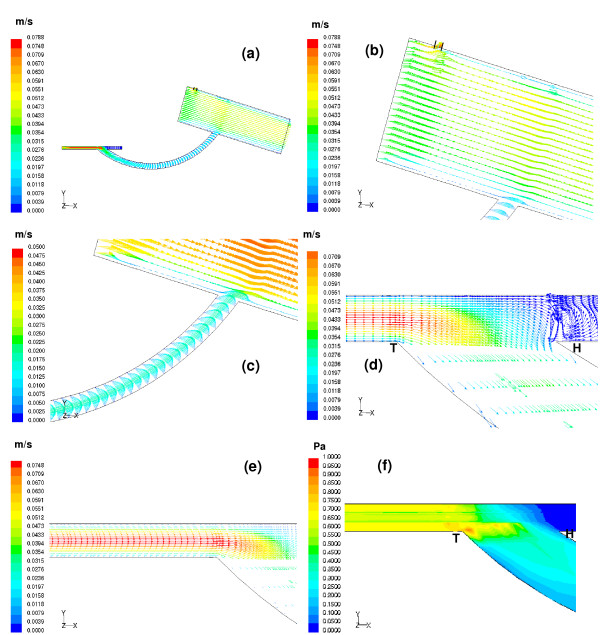
(a) Velocity vectors computed at t = 0.13 sec are depicted on the centre plane of the aorto-left coronary bypass model. (b) The aortic valve is opened and blood flows from the left ventricle down into the aorta. The flow inside the aorta is almost like an inviscid flow. A small amount of the flow enters the right coronary vessel and the remaining moves towards the ascending aorta. An additional flow into the ascending aorta is due to that coming from the left coronary vessel. (c) There is a change in flow direction in the graft. The reverse flow from the left coronary vessel enters the graft. Parabolic profiles of the velocity vectors are seen inside the graft. (d) Very little amount of flow moves towards the proximal portion of the left coronary vessel as it is 100 % occluded. A small region of weak recirculation is seen close to the occluded site. (e) At the start of ejection, there is a reverse flow that comes from the left coronary artery. This flow arises due to the myocardium which is subjected to the intra-myocardial pressure during early systole. As blood flows through the host artery the flow becomes fully developed, this feature no longer exists once the flow reaches the junction. (f) The low velocity gradients in the distal anstomotic junction exhibit low wall shear stress distribution. The maximum wall shear stress magnitude is 1 Pa.

**Figure 7:**
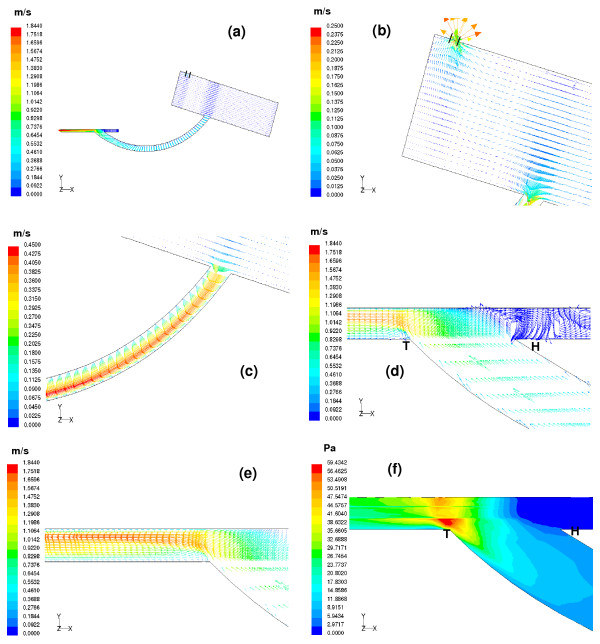
(a) Velocity vectors computed at t = 0.7 sec are depicted on the centre plane of the aorto-left coronary bypass model. (b) The aortic valve remains fully closed at mid-diastole and the flow from the ascending aorta enters both the coronary vessels with a high velocity. (c) Significant skewing of the velocity profiles seen at the outer wall of the graft with a high velocity indicating that good perfusion occurs during mid-diastole. (d) A small amount of flow exiting from the graft moves towards the proximal portion of the bypassed left coronary vessel. As this region is 100% occluded the flow reverses thus giving rise to a recirculation region. (e) Maximum perfusion of the bypassed left coronary vessel occurs during mid-diastole. The peak velocity magnitude is 1.84 m/s seen close to the floor of the host artery. (f) The low velocity components in the vicinity of the occluded region show negligible wall shear stress while elevated shear stresses with a magnitude of 59.43 Pa in the junction region where most of the flow moves towards the distal portion of the left coronary artery.

#### (c) Flow field in the aorto-left coronary bypass model at the onset of ejection, t = 0.13 sec

The distribution of velocity-vectors in the flow field at the onset of ejection, t = 0.13 sec is shown in Figure 6a. The aortic valve opens, and blood flows into the aorta with an entrance uniform velocity of magnitude 0.043 m/s, with some fluid entering the right coronary vessel. The flow inside the aorta behaves like inviscid flow as can be observed in Figure 6b.

During early ejection, as the left coronary vessel is embedded in the myocardium, the myocardium is subject to intramyocardial pressure which causes some reverse flow. The effect of LV contraction on the graft flow is incorporated by adopting the measured flow profile in the left coronary vessel at a point prior to its anastomosis with graft. In this way, we simulate the reversal of flow in the graft at the onset of ejection.

A parabolic velocity profile of the back-flow in the graft is seen in Figure 6c. Most of the fluid flows into the graft; a small amount goes towards the heel of the anastomosis, which results in a weak recirculation region at the occluded region of the bypassed left coronary vessel, as demonstrated in Figure 6d. The back-flow in the distal portion of the left coronary vessel (coming into the anastomosis domain) gets fully developed as it approaches the anastomosis domain, with a maximum velocity of magnitude 0.074 m/s seen along the centreline of the vessel as shown in Figure 6e. The low velocity gradients in the distal anastomotic junction result in low wall shear stress, with maximum magnitude of 1 Pa, as shown in Figure 6f.

#### (d) Flow field in the aorto-left coronary bypass model at the mid-diastolic instant, t = 0.7 sec

The velocity vectors plots at mid-diastole are displayed in Figure 7a. The aortic valve remains closed, and the flow entering the coronaries is caused by the back flow from the ascending aorta as shown in Figure 7b. Most of the fluid enters the graft, exhibiting significant skewing with high velocity magnitude, resulting in maximum perfusion of the host artery as depicted in Figure 7c.

In the anastomosis domain, a strong recirculation region is seen in the heel region of the anastomosis at the distal end of the bypassed vessel, Figure 7d. Maximum perfusion occurs during the mid-diastole phase. The velocity distribution in the perfused host artery segment shows significant skewing towards the floor of the artery (Figure 7e), with a peak velocity magnitude of 1.84 m/s.

In the anastomosis heel region (in the occluded arterial segment) there is negligible shear stress. However, elevated shear stress (of magnitude 59.43 Pa) is seen in the anastomotic toe region (as shown in Figure 7f), where most of the flow moves towards the distal segment of the host coronary vessel.

## Discussion

### Model Geometry

Our model differs from other models in the following ways: (i) both the proximal and distal anastomotic regions are included, while other works [8,9,11-13,15,16,18] have emphasized the distal anastomotic site alone, (ii) the dimensions taken up are close to true dimensions at surgery, unlike the idealized geometries cited in previous investigations[11-13,18] that had all vessels of equal diameters, (iii) the anastomosis geometry is also more realistic compared to the idealized junction angles adopted in previous works, and (iv) our model incorporates the varying cross-section of the graft, that alters gradually along its length from a circular to an elliptical shape in order to fit the smaller artery at the distal anastomosis (thus attempting to reproduce the same geometry as obtained with the surgical procedure).

### Flow patterns

Our results for the aorto-right coronary bypass model and the aorto-left coronary bypass model at two different instants of the cardiac cycle, clearly reveal the following features: (i) at the onset of ejection, in the aorto-right coronary bypass model, very little flow enters the graft with a velocity magnitude around 0.0495 m/s; the maximum flow velocity inside the graft is around 0.2 m/s; (ii) at the mid-diastolic instant, the flow profile in the graft is skewed towards the outer wall, with the peak velocity increasing as it travels downstream; close to the graft exit, the maximum flow velocity attained is around 1 m/s; (iii) at the onset of ejection, in the aorto-left coronary bypass model, there is a backflow from the left coronary artery into the graft; the peak velocity of flow at the entrance to the graft is 0.0788 m/s, and very little perfusion is given to the host artery; (iv) at the mid-diastolic instant, the graft perfusion is maximum, with peak velocity magnitude of 1.1 m/s.

### Features

Our study confirms that blood flow through the coronary artery bypass graft primarily occurs only during the diastolic phase of the cardiac cycle. This is in agreement with the physiological observation of coronary blood flow. There is however some difference between the flow patterns in the right and left coronary graft at the onset of ejection, with some backflow from the left coronary artery into the bypass graft which is not obtained in the case of the right coronary arterial bypass. The phenomenon (of reversal of flow during systole) can be explained by the predominant intra-cardiac course of the left coronary artery system. This aspect is also an original feature of our work.

Lastly, our study has also shown (i) a low wall shear-stress region near the heel region of the anastomosis domain, and (ii) a high wall shear-stress in the toe region of the anastomosis domain, making it prone to intimal hyperplasia. This may have some clinical significance. We should take this into consideration in designing a coronary anastomotic device, so as to minimize biomechanical injuries to the coronary arterial wall. In doing so, we can alleviate or retard the development of intimal hyperplasia, which is the Achilles heel of the effectiveness of coronary artery surgery with saphenous vein.

## Conclusion

The computed results have revealed that (i) maximum perfusion of the occluded artery occurs during mid-diastole, and (ii) the maximum wall shear stress variation was observed around the toe of the anastomotic region. According to our cardiac surgeon joint author (TYS), this preliminary result can enable the clinicians to have a better understanding of vein graft disease, and hopefully we can offer a solution to alleviate or delay the occurrence of vein graft disease.

## Authors' contributions

MS carried out the computational fluid dynamic studies and drafted the manuscript. DNG and LPC guided the study, helped in interpretation of results and critically reviewed the manuscript. YST provided the surgical aspect of the study, and the dimensions for the model. All authors have read and approved the final manuscript.
